# Dose-response risks of all-cause, cancer, and cardiovascular disease mortality according to sex-specific cigarette smoking pack-year quantiles

**DOI:** 10.18332/tid/189952

**Published:** 2024-07-10

**Authors:** Jieun Hwang, Suyoung Jo, Eunsil Cheon, Heewon Kang, Sung-il Cho

**Affiliations:** 1 Department of Health Administration, College of Health Science, Dankook University, Cheonan, Republic of Korea; 2 Institute of Convergence Healthcare, Dankook University, Cheonan, Republic of Korea; 3 Institute of Health and Environment, Seoul National University, Seoul, Republic of Korea; 4 Graduate School of Public Health, Seoul National University, Seoul, Republic of Korea

**Keywords:** smoking, mortality, death, cessation, risk

## Abstract

**INTRODUCTION:**

This study investigated the risks for all-cause death and death from cancer or cardiovascular diseases due to smoking status and behavior, focusing on differences in smoking duration and amount stratified by sex.

**METHODS:**

The integrated Korean Genome and Epidemiology Study provided data for 209770 individuals who were classified as never, former, or current smokers, based on their current smoking status. Pack-years were computed using daily average smoking amount and total smoking duration, and were categorized into quantiles separately for men and women. Based on the number of deaths in 2018, hazard ratios (HRs) were estimated for all-cause mortality, as well as for death caused by all cancers, lung cancer, and cardiovascular diseases according to pack-years adjusted for age, household income, marital status, body mass index, physical activity, and alcohol consumption.

**RESULTS:**

A significant increase in the risk of all-cause mortality was observed for current smokers (men HR=1.90; 95% CI: 1.69–2.14; women HR=2.25; 95% CI: 1.68–2.99) and former smokers (men HR=1.31; 95% CI: 1.17–1.47; women HR=2.35; 95% CI: 1.63–3.39) compared with that for those who had never smoked. Among men, HR for death from lung cancer was 3.13 (95% CI: 2.06–4.75) in former smokers and tended to increase with each pack-year quantile (range HR: 5.72–17.11). Among women, the HR was estimated to be 17.20 (95% CI: 6.22–47.57) only for >3rd quantile.

**CONCLUSIONS:**

Smoking increases the risks of all-cause death. Considering the persistent risks post-smoking cessation, it is vital to focus on preventing smoking initiation and providing proactive support for successful smoking cessation and maintenance of a smoke-free lifestyle.

## INTRODUCTION

Although tobacco use is decreasing worldwide, the difference in smoking rates in low- and high-income countries remains large, and the decrease in smoking rates among women has slowed^1^. Further, approximately 8 million people die worldwide due to smoking^2^. Unless stronger policies for tobacco control are implemented, the number is expected to continue to rise^2^.

The current smoking rate in South Korea has improved considerably, and the decreasing trend is steeper compared with other countries^3^. The smoking rate among men has more than halved from 79.3% in 1980 to 34.0% in 2020, while the smoking rate for women also decreased from 12.6% to 6.6%^4,5^. This reduction is presumably a consequence of various multifaceted and continuous policies (i.e. cigarette price increases, expansion of smoke-free areas, provision of smoking cessation services, and introduction of graphic health warnings) as well as the effect of education and media campaigns to inform the public of smoking-associated health risks^6^. However, the smoking rate among men in South Korea remains above the average of OECD countries; thus, stronger comprehensive policies than the current tobacco control regulation are needed^3^.

Various epidemiological studies have confirmed that smoking is directly and indirectly linked to major causes of death, such as cancer, ischemic heart disease, stroke, and chronic obstructive pulmonary disease (COPD)^7^. Simultaneously, smoking is associated with rising steeply healthcare costs^8^. Hence, smoking causes early death and productivity loss, inducing serious socioeconomic damage worldwide, which was estimated to be over 422 billion US$^9^.

The time taken for smoking to cause disease varies depending on the illness but is estimated to be approximately 20–30 years^10^. The time for disease onset can also vary according to several factors, including smoking amount and duration, age of smoking initiation, extent of inhalation, and cigarette type^11-14^. Furthermore, geography and race also seem to affect these factors^15^. Given the powerful impact of smoking duration and intensity on the development of illnesses, some epidemiological studies use pack-years of cigarette smoking (PCS)^16-18^. PCS are computed by multiplying daily average smoking amounts and cumulative smoking duration, allowing to document individual smoking amounts; moreover, the smoking duration can be intuitively observed.

Previous studies have consistently shown that the hazard ratio (HR) of lung cancer, ischemic heart diseases, aortic valve stenosis, and other illnesses increase as PCS increase^16-20^. However, follow-up studies on mortality of various illnesses due to smoking have limitations, such as insufficient follow-up duration and few deaths. Further, a detailed analysis of smoking duration and amount has never been performed, and only the risks of death by smoking status have been compared. Even among studies in which PCS were used, a persistent limitation is that sex differences in PCS were not considered. Furthermore, apart from sex and age, other personal factors that may affect the risk of mortality, such as drinking and physical activity, were not sufficiently evaluated, and target illnesses were limited to such specific ones as lung cancer and heart diseases, making it impossible to compare the risk across various illnesses.

Accordingly, the present study aimed to estimate the risks of all-cause death and death from each of the major illnesses, due to smoking based on a longitudinal cohort follow-up study conducted in South Korea. The main purpose was to examine differences in the risks of all-cause death and death by lung cancer using PCS, in which smoking amount and duration were factored. The secondary purposes were to accurately assess the risk of smoking in South Korea and present evidence for the need to increase awareness of these risks and promote tobacco regulation policies and promotional education.

## METHODS

### Data and measurements

Data were acquired from the Korean Genome and Epidemiology Study (KoGES) of Korea Disease Control and prevention Agency (KDCA). In KoGES, the general population aged 40–69 years was targeted to collect epidemiological data as well as biospecimens, including blood, urine, and DNA by conducting a large-scale survey on health and lifestyle habits and performing health screening^21^. Specifically, KoGES aims to develop public health and biological indices for chronic diseases currently prevalent among Koreans (such as diabetes, hypertension, obesity, metabolic syndrome, hypercholesterolemia, osteoporosis, and cardiovascular diseases) and to identify disease risk factors^21^.

There are 6 cohorts in KoGES, which are KoGES_ Ansan and Anseong study, KoGES_HEXA (health examinee) study, KoGES_CAVAS (cardiovascular disease association study), KoGES_twin and family study, KoGES immigrant study, and KoGES_emigrant study^21^. In this study, integrated KoGES data were used. Integrated KoGES data refer to a dataset consisting of 201 variables created from common survey items in the KoGES cohorts targeting the general population, i.e. KoGES Ansan and Anseong (n=10030), KoGES_HEXA study (n=173345), and KoGES_CAVAS (n=28338) cohorts^21^.

The dataset contains four questions regarding smoking followed by the available answers: ‘Have you ever smoked?’ (1= ‘No’, 2= ‘I smoked formerly’, 3= ‘I currently smoke’); ‘How old were you when you quit smoking?’ (I stopped smoking before the age of)’; ‘In total, how long have you smoked? (years/months)’; and ‘On average, how many cigarettes did you smoke per day? (approximately)’. Using these questions, smoking status was categorized as never smoker, former smokers, or current smokers. The classification of smokers was considered only at baseline, upon entry into the cohort.

The following sociodemographic participant characteristics were analyzed: sex (men, women), age group (40–49, 50–59, ≥60 years), household income in million KRW(<0.5, 0.5–1, 1–1.5, 1.5–2, 2–3, 3–4, 4–6, >6), marital status (unmarried, married, divorced/separated/widowed), BMI (underweight, normal, overweight, obese), alcohol consumption (non-drinker, ever drinker, current drinker), and physical activity (yes, no).

In this study, the causes of deaths were classified according to the International Classification of Diseases, 10th version (ICD-10). The endpoint was mortality from all-causes, cancer (ICD-10 codes C00-C97), lung cancer (C33-C34), and cardiovascular diseases (I00-I99).

### Data analysis

The KoGES study collected data from 2001 to 2014, gathering a total of 245300 participant records. For this analysis, we excluded individuals with missing data, resulting in a final sample of 209770 participants. The status, date, and cause of death were tracked from 2001 to 2019.

To investigate participants’ general characteristics, the chi-squared test was performed. To examine the characteristics regarding smoking history, descriptive statistics were used to calculate the participants’ age (years) at smoking cessation and its duration (years). Smoking intensity was defined as daily average smoking amount (cigarettes/day) and pack-years [(average number of cigarettes smoked per day/20) × total number of years the person smoked].

To estimate the smoking-related death risk, multivariable-adjusted Cox proportional hazard regression was performed to estimate hazard ratios (HRs) and 95% confidence intervals (CIs) of current and former smokers compared with individuals that have never smoked. To estimate the risk according to smoking amount in detail, current smokers were categorized into quantiles according to the cumulative amount of smoking to estimate the HR of all-cause mortality and death from all cancer, lung cancer, cardiovascular diseases by sex with never smokers as the reference. The analysis adjusted for covariates including age, household income, marital status, alcohol consumption, body mass index (BMI, kg/m^2^), and physical activity. Model 1 included only age as a covariate, while Model 2 included age, household income, marital status, BMI, physical activity, and alcohol consumption.

Data analyses were performed using SAS 9.4 (SAS Institute Inc., Cary, NC, USA), with a statistical significance level of set at p<0.05.

## RESULTS

The demographic characteristics of the study participants are presented in Table 1. Among the 209770 participants, 74379 (35.46%) were men and 135391 (64.54%) were women. The proportion of individuals who did not drink alcohol and performed physical exercise was higher among women (20.5% and 44.2% in men, and 67.0% and 50.1% in women, respectively). The proportion of obesity was higher among men (39.5% in men and 31.6% in women, respectively). The proportion of never, former, and current smokers was 27.5%, 39.3%, and 33.2% in men and 96.2%, 1.3%, and 2.5% in women, respectively. There was a statistically significant sex difference in the proportions of all categories mentioned (p<0.0001).

**Table 1:** Baseline characteristics of study participants by sex (N=209770)

*Characteristics*	*Categories*	*Men (N=74379)*		*Women (N=135391)*		*p*
		*n*	*%*	*n*	*%*	
**Age** (years)	40–49	24239	32.6	49047	36.2	<0.0001
	50–59	25743	34.6	52034	38.4	
	≥60	24397	32.8	34310	25.3	
**Age** (years), mean ± SD		54.64 ±9.10		53.35 ± 8.52		
**Household income** (million KRW)	<0.5	4400	5.92	10762	7.95	<0.0001
	0.5–1	4190	5.63	9263	6.84	
	1–1.5	5990	8.05	11673	8.62	
	1.5–2	7009	9.42	11952	8.83	
	2–3	13477	18.1	22795	16.8	
	3–4	11823	15.9	19179	14.2	
	4–6	9652	13.0	15153	11.2	
	>6	5301	7.13	7112	5.25	
**Marital status**	Unmarried	1657	2.23	2549	1.88	<0.0001
	Married	69090	92.9	113374	83.7	
	Divorced, separated, widowed	3254	4.37	18831	13.9	
**BMI** (kg/m^2^)	Underweight	1182	1.59	2606	1.92	<0.0001
	Normal	21958	29.5	53856	39.8	
	Overweight	21567	29.0	35616	26.3	
	Obese	29345	39.5	42768	31.6	
**Alcohol consumption**	Non-drinker	15264	20.5	90684	67.0	<0.0001
	Former drinker	6274	8.44	3249	2.4	
	Current drinker	52740	70.9	41134	30.4	
**Physical activity**	Yes	32873	44.2	67776	50.1	<0.0001
	No	36598	49.2	62128	45.9	
**Smoking status**	Never smoker	20462	27.5	130276	96.2	<0.0001
	Former smoker	29198	39.3	1802	1.3	
	Current smoker	24719	33.2	3313	2.5	

SD: standard deviation. KRW: 1 million Korean Won about US$730.

In former smokers, both mean smoking duration [23.0 (SD=11.0) in men, 13.7 (SD=10.2) in women] and smoking intensity per day [18.2 (SD=10.5) in men, 9.7 (SD=8.4) in women] were higher in men than in women (p<0.0001) (Table 2). PCS were higher in men [20.6 (SD=17.3)] than in women [6.3 (SD=8.2)] (p<0.0001). Similarly, in current smokers, smoking duration [29.4 (SD=9.9) in men, 17.8 (SD=10.8) in women], smoking intensity [17.6 (SD=8.8) in men, 10.0 (SD=6.7) in women], and PCS [25.0 (SD=15.8) in men, 9.2 (SD=9.6) in women] were higher in men than in women (p<0.0001).

**Table 2:** Smoking history (self-reported) of study participants by sex

	*Men*						*Women*						p*
	*n*	*Mean*	*SD*	*Q1*	*Q2*	*Q3*	*n*	*Mean*	*SD*	*Q1*	*Q2*	*Q3*	
**Former smoker**													
Age of quit (years)	3664	46.2	12.3	39	45	55	186	49.8	14.6	40	49.5	61	0.0013
Duration of smoking (years)	27604	23.0	11.1	15	22	30	1515	13.7	10.2	5	10	20	<0.0001
Intensity of smoking (cigarettes/day)	27728	18.2	10.5	10	20	20	1513	9.7	8.4	4	10	10	<0.0001
Pack-years	15801	20.6	17.3	9	17	28.8	888	6.3	8.2	1.2	3.2	9	<0.0001
**Current smoker**													
Duration of smoking (years)	24017	29.4	9.9	22	29	36	3118	17.8	10.8	10	17	25	<0.0001
Intensity of smoking (cigarettes/day)	24348	17.6	8.8	10	20	20	3213	10.0	6.7	5	10	13	<0.0001
Pack-years	13657	25.0	15.8	15	22.5	32	1784	9.2	9.6	2.5	6	12.5	<0.0001

* The t-test p-values reflect the difference in means between men and women. SD: standard deviation. Q1: 1st quantile. Q2: 2nd quantile. Q3: 3rd quantile.

The estimated HRs of death associated with smoking status and PCS in men are shown in Table 3. Except for total death from cardiovascular disease, all-cause deaths, total cancer death, and lung cancer death showed a statistically significant increase in the HR of former smokers and current smokers compared to never smokers. Moreover, the HR for death in men tended to increase with each increase in quantile of PCS. In Model 2, the HR for all-cause death in former smokers was 1.31 (95% CI: 1.17–1.47, p<0.05) compared to never smokers and increased to 2.70 (95 % CI: 2.16–3.38, p<0.05), 2.76 (95% CI: 2.19–3.49, p<0.05), 2.88 (95% CI: 2.33–3.56, p<0.05), and 3.07 (95% CI: 2.59–3.65, p<0.05) as the quantile of PCS rose by 1. The HR for death due to lung cancer in men tended to increase with each increase in quantile of PCS [Q1–Q2 quantile: 5.72 (95% CI: 2.58–12.69, p<0.05), Q2–Q3 quantile: 8.85 (95% CI: 4.66–16.83, p<0.05) and >Q3 quantile: 17.11 (95% CI: 10.72–27.31, p<0.05].

**Table 3:** Hazard ratios (HR) and 95% confidence intervals (95% CI) for mortality of cause of death by baseline smoking history and pack-years of cigarette smoking in Korea men

*Variables*		*Smoking history*			*Pack-years*					
		Never smoker*	*Former smoker*	*Current smoker*	Never smoker*	*Former smoker*	*<Q1 (<15)*	*Q1–Q2 (15–22.5)*	*Q2–Q3 (22.6–32)*	*>Q3 (>32)*
		*HR (95% CI)*	*HR (95% CI)*	*HR (95% CI)*	*HR (95% CI)*	*HR (95% CI)*	*HR (95% CI)*	*HR (95% CI)*	*HR (95% CI)*	*HR (95% CI)*
**All-cause mortality**	Model 1^a^	1	**1.32 (1.20–1.44)**	**1.92 (1.75–2.11)**	1	**1.32 (1.20–1.44)**	**2.39 (1.99–2.88)**	**2.43 (2.00–2.96)**	**2.51 (2.09–3.00)**	**2.53 (2.20–2.90)**
	Model 2^b^	1	**1.31 (1.17–1.47)**	**1.90 (1.69–2.14)**	1	**1.32 (1.18–1.48)**	**2.70 (2.16–3.38)**	**2.76 (2.19–3.49)**	**2.88 (2.33–3.56)**	**3.07 (2.59–3.65)**
**Cancer mortality**	Model 1^a^	1	**1.62 (1.40–1.88)**	**2.38 (2.05–2.76)**	1	**1.62 (1.40–1.88)**	**2.70 (2.02–3.61)**	**2.35 (1.70–3.26)**	**2.91 (2.20–3.86)**	**3.66 (2.98–4.50)**
	Model 2^b^	1	**1.71 (1.43–2.04)**	**2.47 (2.05–2.97)**	1	**1.73 (1.44–2.06)**	**2.96 (2.08–4.22)**	**2.45 (1.63–3.67)**	**3.37 (2.42–4.69)**	**4.49 (3.50–5.76)**
**Lung cancer mortality**	Model 1^a^	1	**2.82 (1.99–3.99)**	**6.37 (4.54–8.93)**	1	**2.82 (1.99–3.99)**	**3.32 (1.60–6.89)**	**4.21 (2.10–8.47)**	**6.60** (3.76–11.60)	**13.86 (9.44–20.36)**
	Model 2^b^	1	**3.10 (2.05–4.70)**	**6.89 (4.55–10.42)**	1	**3.13 (2.06–4.75)**	2.58 (0.90–7.42)	**5.72 (2.58–12.69)**	**8.85 (4.66–16.83)**	**17.11 (10.72–27.31)**
**CVD mortality**	Model 1^a^	1	1.03 (0.83–1.28)	**1.73 (1.40–2.15)**	1	1.03 (0.83–1.28)	**2.07 (1.31–3.28)**	**2.15 (1.34–3.46)**	**3.37 (2.32–4.92)**	**2.05 (1.46–2.89)**
	Model 2^b^	1	0.91 (0.69–1.21)	**1.74 (1.32–2.30)**	1	0.91 (0.69–1.21)	**2.55 (1.50–4.35)**	**2.88 (1.70–4.89)**	**3.79 (2.42–5.92)**	**2.38 (1.54–3.67)**

a Model 1: adjusted for age.

b Model 2: adjusted for age, household income, marital status, BMI, physical activity, and alcohol consumption. Q1: 1st quantile. Q2: 2nd quantile. Q3: 3rd quantile. CVD: cardiovascular disease. Boldface indicates statistical significance at p<0.05.

* Never smoker: reference.

The HRs estimated of death associated with smoking status and PCS in women are shown in Table 4. Like men, there was a statistically significant increase in the HR of former smokers and current smokers compared to never smokers. In Model 2, HR for all-cause death in female former smokers compared to never smokers was 2.34 (95% CI: 1.63–3.38, p<0.05), and the corresponding HR was 2.54 (95% CI: 1.14–5.68, p<0.05) in the Q1–Q2 quantile group and 5.44 (95% CI: 3.26–9.09, p<0.05) in the >Q3 group compared to never smokers.

**Table 4:** Hazard ratios (HR) and 95% confidence intervals (95% CI) for mortality of cause of death by baseline smoking history and pack-years of cigarette smoking in Korea women

*Variables*		*Smoking history*			*Pack-years*					
		Never smoker*	*Former smoker*	*Current smoker*	Never smoker*	*Former smoker*	*<Q1 (<2.5)*	*Q1–Q2 (2.5–6)*	*Q2–Q3 (6–12.5)*	*>Q3 (>12.5)*
		*HR (95% CI)*	*HR (95% CI)*	*HR (95% CI)*	*HR (95% CI)*	*HR (95% CI)*	*HR (95% CI)*	*HR (95% CI)*	*HR (95% CI)*	*HR (95% CI)*
**All-cause mortality**	Model 1^a^	1	**1.62(1.24–2.12)**	**1.90 (1.55–2.31)**	1	**1.62 (1.24–2.12)**	**2.16 (1.08–4.34)**	1.81 (0.90–3.62)	**2.09 (1.08–4.02)**	**3.46 (2.29–5.23)**
	Model 2^b^	1	**2.35 (1.63–3.39)**	**2.25 (1.68–2.99)**	1	**2.34 (1.63–3.38)**	2.37 (0.98–5.73)	**2.54 (1.14–5.68)**	1.93 (0.80–4.64)	**5.44 (3.26–9.09)**
**Cancer mortality**	Model 1^a^	1	1.42(0.90–2.24)	**1.70 (1.23–2.35)**	1	1.42 (0.90–2.24)	2.19 (0.82–5.86)	0.95 (0.24–3.82)	1.52 (0.49–4.74)	**3.96 (2.18–7.20)**
	Model 2^b^	1	**2.27 (1.35–3.80)**	**2.08 (1.37–3.14)**	1	**2.26 (1.35–3.78)**	2.38 (0.76–7.45)	1.47 (0.37–5.92)	0.72 (0.10–5.14)	**6.78 (3.61–12.71)**
**Lung cancer mortality**	Model 1^a^	1	1.51 (0.56–4.087)	**3.04 (1.72–5.37)**	1	1.51 (0.56–4.08)	0	2.64 (0.37–18.99)	0	**7.4 (2.72–20.18)**
	Model 2^b^	1	1.88 (0.460–7.69)	**4.03 (1.92–8.47)**	1	1.88 (0.46–7.71)	0	4.83 (0.66–35.12)	0	**17.20 (6.22–47.57)**
**CVD mortality**	Model 1^a^	1	1.52 (0.89–2.60)	**2.45 (1.70–3.52)**	1	1.51 (0.88–2.59)	**4.17 (1.33–13.05)**	1.19 (0.17–8.52)	**3.19 (1.02–9.95)**	**4.80 (2.26–10.20)**
	Model 2^b^	1	**2.96** (1.30–6.75)	**3.88 (2.18–6.92)**	1	**2.93** (1.29–6.69)	**8.50 (2.08–34.77)**	3.57 (0.50–25.65)	**7.33 (2.31–23.32)**	**5.20 (1.28–21.10)**

a Model 1: adjusted for age.

b Model 2: adjusted for age, household income, marital status, BMI, physical activity, and alcohol consumption. Q1: 1st quantile. Q2: 2nd quantile. Q3: 3rd quantile. CVD: cardiovascular disease. Boldface indicates statistical significance at p<0.05.

* Never smoker: reference.

## DISCUSSION

This study investigated the risk of death due to smoking using cohort data in South Korea, with smoking status and PCS measures. The increased risk of all-cause mortality in current and former smokers was once again confirmed in this study. We observed increased risks among both men and women. Moreover, increases in lung cancer mortality by PCS were found among former and current male smokers. In particular, the risks increased with each pack-year quantile for men, while PCS for the Q1–Q2 quantile and the Q3 quantile were significant for women. The risks were significant even with considerations of covariates including age, income, marital status, BMI, physical activity, and alcohol drinking. Some increases in the estimates were observed for cardiovascular diseases for different smoking statuses and pack-year quantiles for both sexes.

Our results indicating that former and current smoking increases all-cause deaths corroborate previous findings. An evaluation of 89 cohort studies published since 2015 estimated that the risk for all-cause death increased by 1.55 times in smokers versus non-smokers and that the risk was reduced in people who quit smoking compared to current smokers, increasing only by 1.19 times. Among workers aged 20–85 years who participated in the Japan Epidemiology Collaboration on Occupation Health Study, compared to never smokers, the HR was 1.27 times higher in former smokers and 1.49 times higher in current smokers^22^. Among individuals aged 30–89 years who participated in the Norway Tromsø study, relative to never smokers, the HR increased by 1.18 and 2.05 times in former smokers and current daily smokers, respectively^23^.

In this study, the HR for all-cause death increased in all former and current male or female smokers. For men, the risk for all-cause death increased progressively from never smokers to former and current smokers. Among women, the risk was greater in former smokers than in current smokers. This finding aligns with a previous research form Bangladesh, showing a higher risk for all-cause death in former smokers compared to current smokers^24^. Conversely, in the US, the risk was higher for current smokers than for former smokers^25^. This trend of greater risk in former smokers compared to current smokers in mainly observed in some Asian women^26^. Yang et al.^26^ conducted a study based on data of 16 cohorts in China, Japan, South Korea, Singapore, India, and Bangladesh, and found a higher risk in both former and current smokers compared to never smokers. A separate study on Korean women also showed a higher relative risk in former smokers than in current smokers with lower smoking history, likely due to imbalance in the numbers of former, current, and never smokers^27^. Therefore, while the sample size of women in this dataset was large, the proportions of former and current smokers were relatively small at 1.3% and 2.5%, respectively, and results should be interpreted with caution.

Additionally, our results indicated that the HR for all-cause death among former smokers was greater in women than men. Further, the corresponding HR among current smokers was greater in women than men. Greater HR for all-cause death in women compared to men suggests that women may be more susceptible to death due to smoking^28,29^. The absence of a sex difference in such susceptibility was raised in two studies^30,31^. However, Lariscy et al.^32^ reported that although the level of risk was similar in both sexes, it was higher in women compared to men. Greater HR in women may be explained by stronger associations with exposure to smoke during cooking, secondhand smoking, and human papillomavirus^33,34^. Likewise, the HR was higher for South Korean women^27^, suggesting the presence of sex differences in susceptibility to death.

Regarding lung cancer, in both sexes, the risk steadily increased in former and current smokers compared to never smokers. Notably, the risk in the current study was greater than those reported in existing domestic studies. In one of the earliest studies in Korea, which targeted public servants and teachers aged >30 years who underwent health screenings between 1992 and 1995, found that male smokers had a significantly higher risk of lung cancer incidence and mortality compared to male non-smokers^11^. Likewise, another study with a similar study population reported a higher risk of lung cancer incidence in current smokers^35^.

Particularly, when PCS were taken into account, the risk increased to a considerable level. In both men and women, the death risk steadily rose as smoking amount increased^36^. When considering only smoking status and pack-years, men had an increased risk for all-cause death, which was higher in those with greater smoking history. Women showed a similar trend, with a significantly higher risk in heavy smokers. For lung cancer, the risk increased substantially in both men and women with higher smoking levels. With PCS taken into account, these risk levels were similar to those observed in a previous study^27^. Grouping pack-years into quantiles, computed separately for men and women, showed that the risks in our study were higher than those previously reported^19^. Given the average pack-years for former and current smokers, it may be challenging to derive solid conclusions by grouping the sexes into the same categories.

Likewise, the current study findings suggest that in explaining smaller HRs reported in studies conducted in Asian countries compared to those conducted in Western countries, which has been pointed out as a limitation, it may be erroneous to classify both sexes into the same categories and not consider differences in PCS among different countries and sexes^26^. So far, smaller HRs have been estimated in Asian countries relative to Western countries due to several factors, including relatively lower levels of smoking amount and duration and CYP2A6 genetic polymorphism^35^. This issue was somewhat resolved here by including PCS and other control variables. Although there are differences in smoking rates between men and women, there has been a tendency to omit sex differences from past analyses^26^. Attempts to analyze differences in health effects based on sex differences have often been attempted, and such attempts need to be continued in order to improve the limitations of studies thus far. Our findings have important implications for understanding the generalizability of smoking-related risks to other Asian populations. The trends observed in our study align with those seen in countries such as Japan and China, where similar increases in all-cause and lung cancer mortality among smokers have been reported^22,27^. These comparisons contextualize our results within a broader Asian framework, suggesting significant commonalities in smoking-related health risks across Asia despite regional differences. However, caution should be exercised when generalizing these findings due to potential variations in cultural, socio-economic, and health policy contexts^37^. Factors such as different smoking habits, public health interventions, and genetic predispositions may influence the outcomes^38^. Future research should include diverse populations from various Asian countries to better understand these differences and similarities.

### Limitations

The study has the following limitations. First are limitations inherent to the KoGES data. Like the target populations of other prospective cohort studies^39^, the study population of KoGES is not a representative sample of the total population of South Korea. Hence, a prospective cohort study has no issues in identifying an exposure-response link, but caution should be taken when generalizing the findings to the total population. Additionally, the KoGES integrated data are combined data of community-based, urban, and rural cohorts out of all KoGES cohorts and, thus, there could be discrepancies among the three cohort surveys. However, the impacts of integrating different cohorts on current study findings are expected to be trivial because the data for three cohorts were collected using standardized procedures^21^. Second, because the data regarding personal history of smoking in the KoGES were collected only in the beginning of the study and possible changes were not followed up, individuals initially identified as smokers continued to be classified as such, even if they quit smoking afterward. Indeed, the risk of death does not decrease during the early period of smoking cessation, but it does decrease as the length of smoking cessation increases^36^. Hence, in this study, the HRs may have been over-estimated if individuals initially classified as smokers quit smoking, and underestimated if former smokers who did not smoke in the beginning started to smoke again. Third, in addition to age, household income, marital status, BMI, alcohol consumption, and physical activity were used as control variables in this study. Aside from these control variables, the risk for death due to smoking can be estimated with considerations of family history and medical history^19,25^, diseases such as hypertension, diabetes, hypercholesterolemia^22,27^, residential area^28^, and education level^26^. In the present study, it was impossible to include all of those variables due to limitations in the data, but future studies should consider them. Fourth, the study did not account for competing risks, which could lead to biased estimates if individuals are censored due to other causes of death that are related to smoking status. Nevertheless, we attempted to mitigate this issue by carefully selecting the study cohort and ensuring thorough follow-up procedures. However, future studies should employ more sophisticated statistical methods to formally account for competing risks.

## CONCLUSIONS

The current findings confirmed increased smoking-related death risk among former and current smokers, and that previously reported lower risks were due to a lack of details for smoking behaviors, such as smoking amount and duration. Specifically, the current results indicate that the risk of death due to smoking increased in women as much as in men, despite low smoking rates among Asian women. Because the risks due to smoking persist even after individuals quit, it is of utmost importance to both prevent the initiation of smoking and provide proactive support and efforts towards encouraging individuals to quit smoking and successfully maintain a non-smoking status.

## Data Availability

The data supporting this research are available from the source: (https://nih.go.kr/ko/main/contents.do?menuNo=300566).
